# Crystallographic insights into diamond-shaped 7M martensite in Ni–Mn–Ga ferromagnetic shape-memory alloys

**DOI:** 10.1107/S2052252519010819

**Published:** 2019-08-15

**Authors:** Zong-Bin Li, Bo Yang, Yu-Dong Zhang, Claude Esling, Xiang Zhao, Liang Zuo

**Affiliations:** a Key Laboratory for Anisotropy and Texture of Materials (Ministry of Education), School of Material Science and Engineering, Northeastern University, Shenyang 110819, People’s Republic of China; b Laboratoire d’Étude des Microstructures et de Mécanique des Matériaux (LEM3),CNRS UMR 7239, Université de Lorraine, Metz 57045, France; c Laboratory of Excellence on Design of Alloy Metals for low-mAss Structures (DAMAS), Université de Lorraine, Metz 57045, France

**Keywords:** Ni–Mn–Ga alloys, martensitic transformation crystallography, twin relationships, electron backscatter diffraction (EBSD)

## Abstract

For Heusler-type Ni–Mn–Ga ferromagnetic shape-memory alloys, the configuration of the martensite variants is a decisive factor in achieving a large magnetic shape-memory effect through field-induced variant reorientation. Based upon the spatially resolved electron backscatter diffraction technique, the microstructural evolution associated with the martensitic transformation from austenite to seven-layered modulated (7M) martensite was investigated on a polycrystalline Ni_53_Mn_22_Ga_25_ alloy. It was clearly shown that grain interior nucleation led to the formation of diamond-shaped 7M martensite within the parent austenite matrix. This diamond microstructure underwent further growth through an isotropic expansion with the coordinated outward movement of four side habit planes, followed by an anisotropic elongation with the forward extension of a type-I twin pair. A two-step growth model is proposed to describe the specific morphology and crystallography of 7M martensite. In addition, the habit planes were revealed to possess a stepped structure, with the {1 0 1}_A_ plane as the terrace and the {0 1 0}_A_ plane as the step. The characteristic combination of martensite variants and the underlying mechanism of self-accommodation in the martensitic transformation have been analysed in terms of the minimum total transformation strain, where the deformation gradient matrix was constructed according to the experimentally determined orientation relationship between the two phases. The present results may deepen the understanding of special martensite microstructures during the martensitic transformation in ferromagnetic shape-memory alloys.

## Abstract 2

The crystallographic features associated with the martensitic transformation from austenite to seven-layered modulated (7M) martensite in Ni–Mn–Ga ferromagnetic shape-memory alloys have been investigated through electron backscatter diffraction (EBSD) measurements.

## 1. Introduction   

The versatile functionalities of shape-memory alloys, which have wide potential applications, stem from the characteristic reversible martensitic transformation under an external field. In general, this type of phase transformation involves the nucleation and growth of martensite in the parent austenite. The phase interface is always the so-called invariant plane or habit plane where the transformation strains are cancelled. Since the product phase has lower crystal symmetry than the parent austenite, several martensite variants can form from the same parent crystal. It has frequently been observed that individual martensite variants in shape-memory alloys are assembled into groups with regular shapes, such as spear (Otsuka & Shimizu, 1974 ▸; Schroeder & Wayman, 1977 ▸), wedge (Balandraud & Zanzotto, 2007 ▸; Bhattacharya, 1991 ▸), triangle (Chai *et al.*, 2009 ▸; Krishnan, 1998 ▸; Miyazaki *et al.*, 1989 ▸), hexangle (Inamura *et al.*, 2012 ▸; Nishida *et al.*, 2012*a* ▸, 2012 ▸ *b*; Soejima *et al.*, 2016 ▸) or diamond (Gao *et al.*, 2000 ▸; Murakami *et al.*, 1994 ▸; Niemann *et al.*, 2017 ▸; Saburi & Wayman, 1979 ▸; Wayman, 1994 ▸). Certainly, the specific morphology and configuration of a variant group depends on the nature of the self-accommodation to minimize the transformation strain energy accompanying the nucleation and growth of the martensite.

Theoretically, the crystallographic features of martensitic transformation can be predicted by the phenomenological theory of martensite crystallography (PTMC) (Cong *et al.*, 2007 ▸; Jin & Weng, 2002 ▸; Wechsler *et al.*, 1953 ▸; Zhu & Liew, 2003 ▸), where the Bain distortion or Bain correspondence is widely used for modelling the deformation gradient and the formed microstructure. It is noted that the Bain correspondence only concerns the geometric correspondence between the initial and final lattices, which describes the overall lattice deformation but not necessarily every atom in the lattice (Bhattacharya, 2003 ▸). Such a mathematical treatment may yield a much higher atomic misfit than the real atomic motion, rendering an energetically unfavourable situation. Indeed, the transformation from austenite to martensite follows a specific orientation relationship, which provides some clues to trace the possible transformation strain paths between the two phases. In this regard, modelling the deformation gradient of the martensitic transformation based on the orientation relationship between the two phases may make a more straightforward correlation with the experimentally observed morphological features and crystallographic orientation, since the in-plane atomic shear is involved.

The Heusler-type Ni–Mn–*X* (*X* = Ga, In, Sn, Sb) alloys represent a novel class of shape-memory alloys (Kainuma *et al.*, 2006 ▸; Karaca *et al.*, 2009 ▸; Li *et al.*, 2016 ▸, 2018 ▸; Li, Dong *et al.*, 2019 ▸; Li, Yang *et al.*, 2019 ▸; O’Handley *et al.*, 2000 ▸; Sozinov *et al.*, 2013 ▸; Zhang *et al.*, 2017 ▸; Zhao *et al.*, 2019 ▸) which combine the properties of ferromagnetism with those of a thermoelastic martensitic transformation. In particular, large magnetic field-induced strains up to ∼12% are achievable in the ferro­magnetic martensite state through magnetically driven rearrangement of variants (O’Handley *et al.*, 2000 ▸; Sozinov *et al.*, 2013 ▸). In contrast with the conventional thermally activated shape-memory alloys, these alloys have a comparable field-induced strain output, but the mode of activation by a magnetic field gives them a much higher response frequency. Thus, they are conceived as potential candidates for the new generation of smart actuator or sensor materials.

The martensitic transformation behaviours of Ni–Mn–Ga alloys are strongly composition dependent (Chernenko, 1999 ▸). Even a slight compositional variation may significantly change the martensitic transformation temperatures and martensite crystal structure. In the literature, the most frequently found are five-layered modulated (5M), seven-layered modulated (7M) and non-modulated (NM) martensite. It has been revealed that the 5M or 7M martensite usually exhibits a monoclinic superstructure (Brown *et al.*, 2002 ▸; Li *et al.*, 2010 ▸, 2011*a* ▸, ▸ *b*; Pons *et al.*, 2000 ▸; Righi *et al.*, 2008 ▸), whereas the NM martensite possesses a tetragonal crystal structure (Pons *et al.*, 2000 ▸). To date, the phase transformation characteristics of Ni–Mn–Ga alloys have been investigated mainly by measuring thermodynamic parameters at a global level, including calorimetry (Jiang *et al.*, 2004 ▸; Wu & Yang, 2003 ▸), thermo-magnetic analysis (Skokov *et al.*, 2012 ▸; Stadler *et al.*, 2006 ▸) and temperature-dependent diffraction (Buschbeck *et al.*, 2009 ▸; Chernenko *et al.*, 1998 ▸; Li, Li *et al.*, 2019 ▸). Although these measurements allow one to determine the critical transformation temperatures as well as the transformation sequences, they do not provide detailed information on local microstructural evolutions nor on crystallographic correlations associated with the phase transformation.

**Figure 1:**
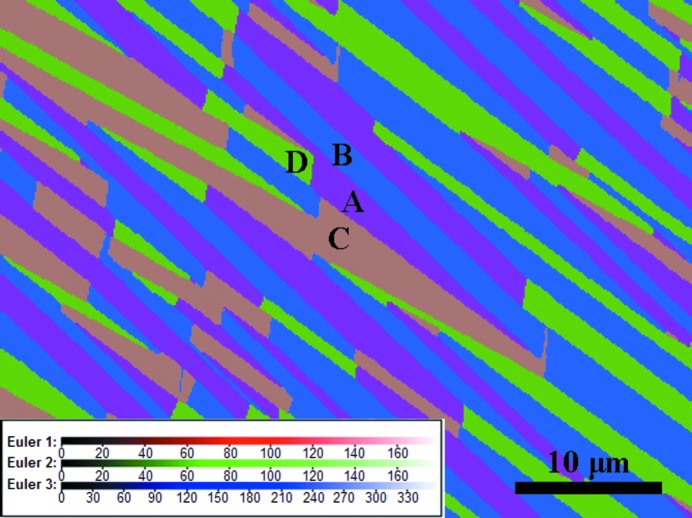
An EBSD ‘all Euler’ orientation micrograph of 7M martensite with four variants (A, B, C and D) in one variant colony in an Ni_50_Mn_30_Ga_20_ alloy. The inset shows the legends of the Euler angles.

Recently, microstructure-correlated crystallographic characterizations were performed on different types of Ni–Mn–Ga martensite by means of electron backscatter diffraction (EBSD) (Chulist *et al.*, 2012 ▸, 2013 ▸, 2016 ▸, 2017 ▸; Cong *et al.*, 2006 ▸, 2007 ▸; Hou *et al.*, 2017 ▸, 2018 ▸; Li *et al.*, 2010 ▸, 2011*a* ▸, ▸ *b*, 2012 ▸, 2013 ▸, 2014 ▸; Scheerbaum *et al.*, 2010 ▸). The excellent orientation examination capacity of modern EBSD systems has enabled the definite determination of the morphology and configuration of self-accommodated martensite, the orientation relationships between adjacent variants, and the character of the interface planes. The 7M martensite exhibits the characteristics of a plate-shaped microstructure, as exemplified in Fig. 1 ▸. It has been revealed that the four local variants in a variant group form three types of twin relationship (Li *et al.*, 2010 ▸), *i.e.* type-I twin (A:C or B:D), type-II twin (A:B or C:D) and compound twin (A:D or B:C). However, it is still not clear what the underlying mechanism is for the formation of this type of microstructure, nor the role of the different types of twin relationship during the martensitic transformation. The corresponding martensitic transformation crystallography and strain accommodation mechanism still need to be explored further.

Undoubtedly, a comprehensive understanding of the morphological and crystallographic features of martensite in Ni–Mn–Ga alloys would give explicit guidelines for property optimization through microstructural control. In particular, a deep insight into the lattice deformation behaviour of the martensitic transformation is of interest because it may provide a possible mechanism by which the transformation can be easily induced. In the present study, a polycrystalline Ni_53_Mn_22_Ga_25_ bulk alloy with the co-existence of cubic austenite and monoclinic 7M martensite at room temperature was chosen as a prototype material. The microstructural evolutions accompanying the martensitic transformation were analysed by EBSD. Moreover, in order to analyse the lattice deformation from austenite to 7M martensite, the deformation gradient was resolved by considering the atomic shear involved in the orientation relationship between the two phases. It was revealed that the shear strain associated with the martensitic transformation can be effectively accommodated by the combination of four twin-related variants in one variant group, whereas the accommodation of principal strain induced by lattice elongation or contraction can be realized through the combination of different variant groups.

## 2. Experimental   

Polycrystalline Ni_53_Mn_22_Ga_25_ (at.%) alloy was prepared by arc melting. The details of the sample preparation can be found elsewhere (Li *et al.*, 2012 ▸). The actual composition was determined to be Ni_53.4_Mn_21.8_Ga_24.8_ using an energy-dispersive spectrometer (EDS) attached to the field-emission gun scanning electron microscope (FEG–SEM, see below). The martensitic transformation temperatures were measured by differential scanning calorimetry (DSC). The start and finish temperatures of the forward and inverse martensitic transformations were determined to be 286.5 K (*M*_s_), 277.9 K (*M*_f_), 289.3 K (*A*_s_) and 303.1 K (*A*_f_) (Li *et al.*, 2012 ▸), respectively. The crystal structures of the constituent phases were examined with powder X-ray diffraction (XRD) using Cu *K_α_* radiation. According to the XRD patterns measured at 298 and 243 K, the alloy has undergone a transformation from austenite to 7M martensite on cooling. The austenite phase has a cubic structure (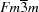, No. 225) with lattice constant *a*_A_ = 5.811 Å, and the 7M martensite has a monoclinic incommensurate superstructure (*P*2/*m*, No. 10) with the lattice constants *a*_7M_ = 4.222 Å, *b*_7M_ = 5.537 Å, *c*_7M_ = 41.982 Å and β = 92.5° (Li *et al.*, 2012 ▸). The microstructural characterizations were carried out using an optical microscope with polarized light and an FEG–SEM (JEOL JSM 6500F) with an EBSD analysis system. The ‘beam-control’ mode was applied for auto EBSD orientation mapping. Detailed microstructures were examined with a JEOL JEM-2100F transmission electron microscope operated at 200 kV.

The twin relationship between neighbouring variants was identified based on misorientation calculations (Li *et al.*, 2011 ▸ *b*). The characteristic interfaces (twin interface between neighbouring variants, and habit plane between austenite and martensite) were analysed using the indirect two-trace method (Zhang *et al.*, 2007 ▸). The Miller indices of individual interface planes were determined according to the orientation data of adjacent crystals and the trace vectors of interfaces in the sample coordinate system. The strain accommodation of various variant combinations was evaluated based on the deformation gradient tensor of individual variants from austenite to martensite. The deformation gradient tensor was constructed by examining the atomic correspondences under the virtual orientation relationship between the two phases.

## 3. Results   

### 3.1. Nucleation of 7M martensite   

**Figure 2:**
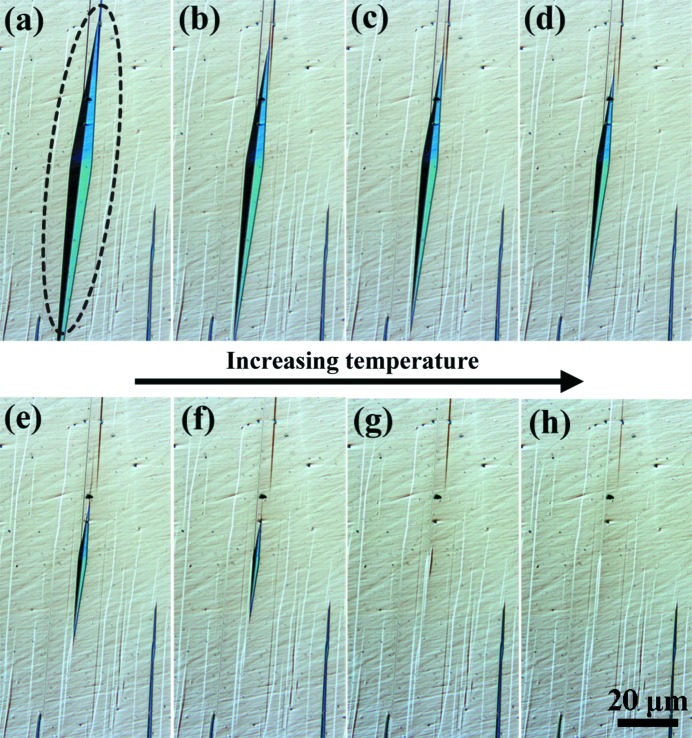
(*a*)–(*h*) Optical micrographs obtained under polarized light, showing the microstructural evolution of diamond-shaped 7M martensite in austenite with increasing temperature.

For the present Ni_53_Mn_22_Ga_25_ alloy, both austenite and 7M martensite phases co-exist at room temperature (298 K), as revealed by the XRD measurements (Li *et al.*, 2012 ▸) and confirmed by the microstructural observations. Fig. 2 ▸ presents a series of optical micrographs that are representative of the microstructural evolution with the inverse martensitic transformation (*i.e.* from 7M martensite to austenite). Here, the sample was pre-cooled to 268 K to be in the full martensite state and then subjected to optical microscopy observations at room temperature. As the austenitic transformation start temperature (*A*_s_ = 289.3 K) was higher than the body temperature of the pre-cooled sample, the inverse martensitic transformation was thermally activated when the body temperature of the sample was elevated.

Since the microstructure was observed in an optical microscope under polarized light, the change in the contrast of the micrographs reflects the crystallographic orientation difference for the same phase and the structure difference for different phases. It is seen from Fig. 2 ▸(*a*) that at a relatively low temperature, the 7M martensite has a diamond-like shape with dark contrast (circled with the black dashed ellipsoid) in an austenite grain with uniform grey contrast. The contrast variation in the martensite diamond reflects the fact that there are different crystallographic orientation variants present. This diamond-like microstructure of 7M martensite was also confirmed by subsequent EBSD measurements, as shown below. With increasing temperature [Figs. 2 ▸(*b*)–2(*h*)], the diamond-shaped martensite gradually shrinks until it dis­appears completely. Since the martensitic transformation in Ni–Mn–Ga alloys is reversible through the forward and backward movements of the phase interfaces (habit planes), the microstructural evolution shown in Fig. 2 ▸ indirectly demonstrates that the martensitic transformation is initiated by forming the diamond-shaped 7M martensite cluster.

**Figure 3:**
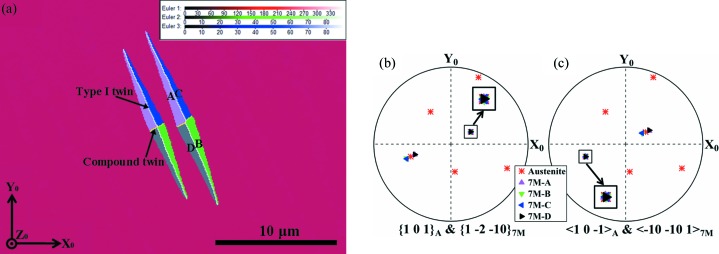
(*a*) An EBSD ‘all Euler’ orientation micrograph of diamond-shaped 7M martensite with four distinct variants (A, B, C and D) in austenite. The inset shows the legends of the Euler angles. (*b*) {1 0 1}_A_ and {1 −2 −10}_7M_ pole figures and (*c*) 〈1 0 −1〉_A_ and 〈−10 −10 1〉_7M_ pole figures of austenite and 7M martensite. The common poles are highlighted by squares in the figures.

To access the detailed microstructural organization and crystallographic features of the diamond-shaped martensite constituents, EBSD orientation characterizations were performed on the co-existing austenite and 7M martensite at room temperature. Fig. 3 ▸(*a*) shows a typical EBSD orientation micrograph containing two sets of diamond-shaped martensite, where the austenite phase and the 7M martensite phase are coloured according to their crystallographic orientations. Clearly, each diamond-shaped martensite consists of four orientation variants (denoted A, B, C and D). Each variant has as neighbours two other variants with the typical type-I (A:C and B:D) and compound (A:D and B:C) twin relations and is bordered by the corresponding twinning planes ({1 −2 −10}_7M_ for the type-I twins and {1 0 10}_7M_ for the compound twins) (Li *et al.*, 2012 ▸), as illustrated in Fig. 3 ▸(*a*). The type-I twin interfaces constitute the longer ridge of the diamond and the compound twin interface the shorter ridge. Thus, the diamond-like 7M martensite microstructure – formed at a very early stage of the martensitic transformation – can be visualized as two spears placed back to back.

It should be noted that the two kinds of twin relationship between the four variants (*i.e.* type-I twin and compound twin) and the corresponding two kinds of twin interface separating the variants in each diamond-shaped cluster have been clearly revealed for the 7M martensite in Ni–Mn–Ga alloys when the martensitic transformation is complete (Fig. 1 ▸). However, the adjacency between variants A and B or C and D that represents the type-II twin combination of variants is absent from the diamond cluster but appears frequently in the final martensite microstructure, as shown in Fig. 1 ▸.

The orientation relationships between the parent austenite and the 7M martensite were determined using the measured EBSD orientation data. The energetically favourable orientation relationship for the austenite to 7M martensite transformation can be referred to as the Pitsch relation, *i.e.* {1 01}_A_||{1 −2 −10}_7M_ and 〈1 0 −1〉_A_||〈−10 −10 1〉_7M_, as demonstrated in Figs. 3 ▸(*b*) and 3 ▸(*c*). As seen in the pole figures, the four 7M variants share one common {1 −2−10}_7M_ and one common 〈−10 −10 1〉_7M_. The common {1 −2 −10}_7M_ and 〈−10 −10 1〉_7M_ correspond to one of the {1 0 1}_A_ planes and one of the 〈1 0 −1〉_A_ directions, respectively. This result is consistent with our previous findings on a polycrystalline Ni_50_Mn_30_Ga_20_ alloy (Li *et al.*, 2011*a* ▸). It demonstrates that such an orientation relationship between austenite and 7M martensite is established at the nucleation stage of the martensitic transformation. Under the above-mentioned lattice parallelism, an orthonormal common coordinate frame along [1 0 −1]_A_–[1 0 1]_A_–[0 −1 0]_A_ can be constructed to make a straightforward correlation between four austenite and 7M variants to describe the lattice deformation from austenite to martensite, as will be discussed below.

### 3.2. Growth of 7M martensite   

By examining the shrinking process of the diamond-shaped 7M martensite group during the inverse martensitic transformation, as shown in Fig. 2 ▸, one can figure out two features. The first is that during the dynamic evolution process, the volume ratio of the four variants remains equal and the orientations of the habit planes stay unchanged. As a consequence of this, the shape of the diamond stays constant although its size changes. The second is that the variant interfaces (the long and short ridges of the diamond or the type-I and compound twin interfaces) do not make any translational movement. This means that the size change of the martensite cluster results merely from the phase transformation without any twinning or detwinning between internal variants. This indirectly shows that, at an early stage of the martensite growth, the initial diamond-shaped martensite expands into the austenite matrix in an isotropic manner. It requires that the four side habit planes bordering the diamond-shaped martensite should all be active and move outwards simultaneously to increase the volume fraction of the martensite. Clearly, in such a manner the martensite variants cannot evolve into a plate shape and the type-II twin relation cannot be formed, but it has been observed in the final microstructure. Thus, a different growth manner must be involved in the subsequent growth process.

**Figure 4:**
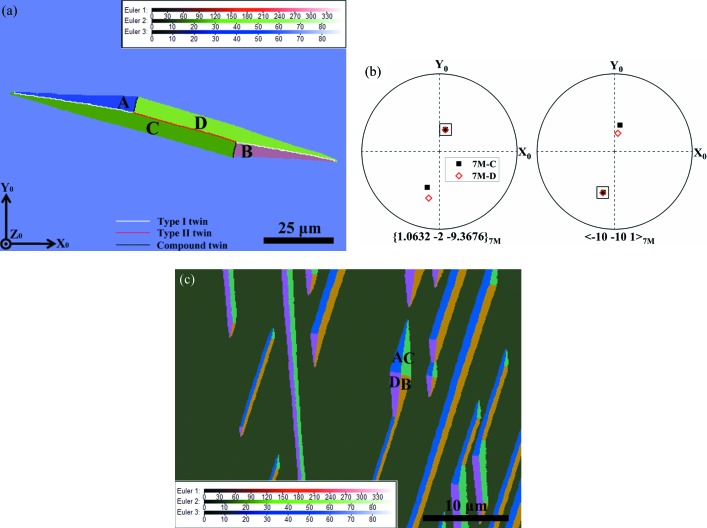
(*a*) An EBSD ‘all Euler’ orientation micrograph showing anisotropic elongation of diamond-shaped martensite. (*b*) Pole figures of (left) the {1.0632 −2 −9.3676}_7M_ twinning plane and (right) the 〈−10 −10 1〉_7M_ twinning direction for variants C and D with a type-II twin. The common poles are highlighted by squares in the figures. (*c*) An EBSD ‘all Euler’ orientation micrograph showing paired martensite plates with a spear head. The inset shows the legends of the Euler angles.

Fig. 4 ▸(*a*) shows a spearheaded plate-shaped martensite cluster with the same group of variants as those in the diamond cluster, indicating a different manner of growth when the size of the diamond cluster reaches a certain limit. The second stage of growth of the martensite cluster from the diamond shape to the spearheaded plate shape is realized by the elongation of the diamond in the length direction through the forward motion of two opposite habit planes [for example the phase interfaces of variants A and B in Fig. 4 ▸(*a*)]. During this motion, only one side habit plane in each spearhead can move into the austenite matrix without increasing the interfacial area, while the other side habit plane is passively dragged to increase the interfacial area. As the area fractions of variants having movable habit planes are constant, the short ridges (the compound twin interfaces between variants A and D or B and C) are dragged apart from their intersection in the diamond agglomerate, accompanied by the introduction of the adjacency of variants C and D, being in a type-II twin relation. Consequently, the two type-I twin interfaces are bridged by the type-II twin interface.

Fig. 4 ▸(*b*) shows the pole figures of {1.0632 −2 −9.3676}_7M_ and 〈−10 −10 1〉_7M_ for variants C and D with a type-II twin, where {1.0632 −2 −9.3676}_7M_ and 〈−10 −10 1〉_7M_ represent the twinning plane and twinning direction, respectively. Detailed twinning elements can be found elsewhere (Li *et al.*, 2012 ▸). It is seen that there are respective common poles for the twinning plane and twinning direction, confirming the type-II twin relation between variants C and D.

**Figure 5:**
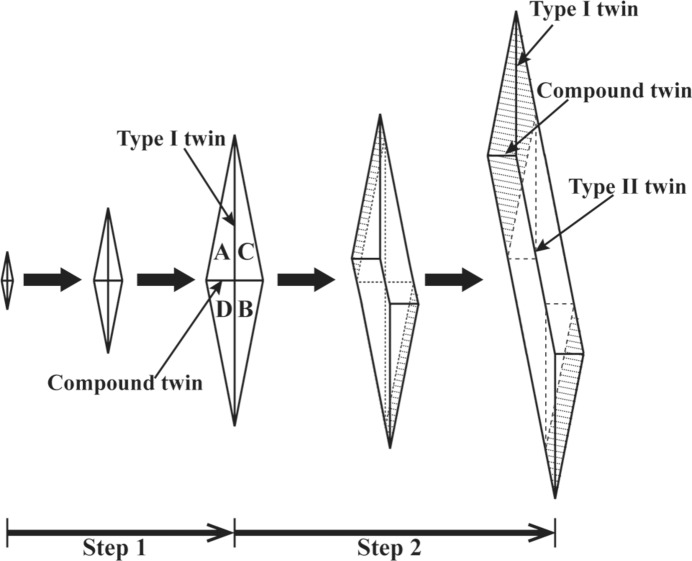
A schematic illustration of the two-step growth of 7M martensite from a diamond shape to a spearheaded plate.

As the movable habit plane always keeps the interfacial area constant, the compound twin interface [interface between A and D or B and C in Fig. 4 ▸(*a*)] and the type-I twin interface [interface between A and C or B and D in Fig. 4 ▸(*a*)] intersecting it should also make a coordinated movement. Thus the newly formed variant A (or B) constantly transforms its volume to variant D (or C) through a compound detwinning process and to C (or D) through a type-I detwinning process. By such coordinated interface motions, including the phase interface motion between austenite and a single variant and the twin interface motion between two variants in compound twin and type-I twin relations, the initially diamond-shaped martensite clusters grow into spearheaded plate-shaped martensite, as shown in Fig. 4 ▸(*c*). The two-step growth process of 7M martensite is illustrated in Fig. 5 ▸.

**Figure 6:**
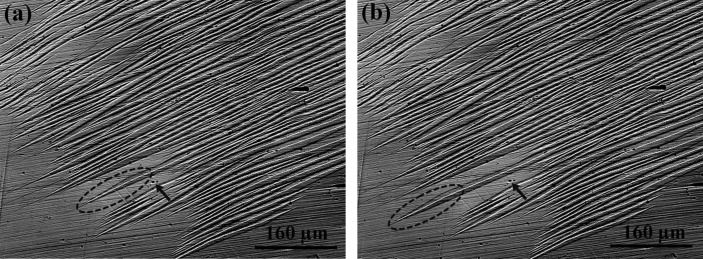
(*a*), (*b*) Optical micrographs showing the forward motion of a spearhead on cooling. The forward spearhead is highlighted with a black dashed ellipsoid.

With the aid of *in situ* microstructural observations, some direct images were obtained to confirm the forward motion of the spearheaded martensite plates during plate growth. Fig. 6 ▸ shows two optical micrographs of 7M martensite plates taken on cooling. Here, the sample was first heated to 303 K and then observed without heat input in an optical microscope. It is clearly seen that, on cooling, the martensite plate was elongated in length through the forward motion of its spearhead, as highlighted with the dashed ellipsoids in Figs. 6 ▸(*a*) and 6 ▸(*b*). The defect on the observed sample (indicated with the arrows in the two figures) serves as a reference to correlate the microstructures in the two figures.

Through the repeated nucleation and growth processes, the volume fraction of 7M increases gradually until the austenite disappears completely. Once the martensitic transformation is completed, individual 7M martensite plates are featured with distinct groups, each containing four differently oriented plate variants that are distributed alternately in a twin relation. These four plate variants correspond to the four orientation variants constituting the initial diamond-like martensite microstructure. It is usually observed that several variant groups form in the same parent austenite grain.

### 3.3. Characteristics of phase interfaces   

The above-mentioned results show that the growth of 7M martensite is realized through the movement of the austenite–martensite interface and the orientation of the phase interface is maintained through the growth process. Here, the macroscopic interface planes between austenite and a single 7M martensite variant were analysed experimentally using the indirect two-trace method (Zhang *et al.*, 2007 ▸). Two independent interface trace vectors in the sample coordinate system from the same kind of interface and the orientation data of their adjacent crystals measured by EBSD were used as the initial input for the interface plane-normal determination. To achieve statistical reliability, 16 habit plane normals were calculated and the calculation results, expressed in the coordinate frame of the austenite lattice, are listed in Table 1 ▸. The mean value of the calculated habit plane normals was determined to be {0.736130, 0.673329, 0.068855}_A_, which is close to {1 1 0}_A_ with 4.7° deviation. In fact, the austenite/martensite interfaces bordering the martensite cluster, either the diamond-shaped or the spearheaded plate-shaped, are of the same family of planes.

**Figure 7:**
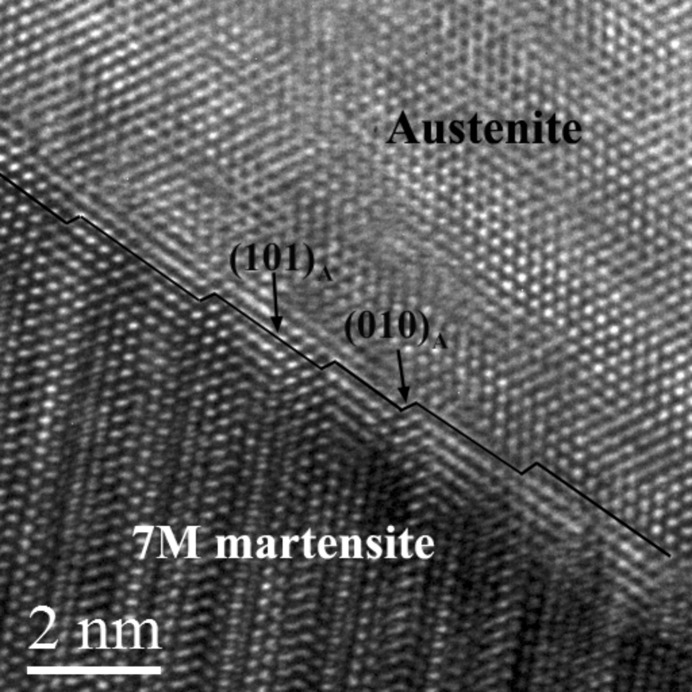
A high-resolution TEM image of the austenite–7M martensite interface. The incident beam is along 〈1 1 −1〉_A_.

The atomic structures of the austenite–martensite interface were further analysed at room temperature. Fig. 7 ▸ shows a high-resolution TEM image of the austenite–7M martensite phase interface, where the incident beam is along the 〈1 1 −1〉_A_ axis (corresponding to the 〈2 1 0〉_7M_ axis). It can be seen that the interface possesses a step structure with the (1 0 1)_A_[||(1 −2 −10)_7M_] plane as the terrace and the (0 1 0)_A_[||(1 0 10)_7M_] plane as the step. The terrace plane is a common plane between the two lattices, which is also termed the atomic habit plane (Ye & Zhang, 2002 ▸). Such a step structure permits an invariant plane without accumulated rotation or distortion at the macroscopic level by a coherent interface at the atomic level, thus securing a low interfacial energy. Owing to the existence of the steps, the macroscopic interface plane deviates from the rational {1 1 0}_A_ plane.

**Figure 8:**
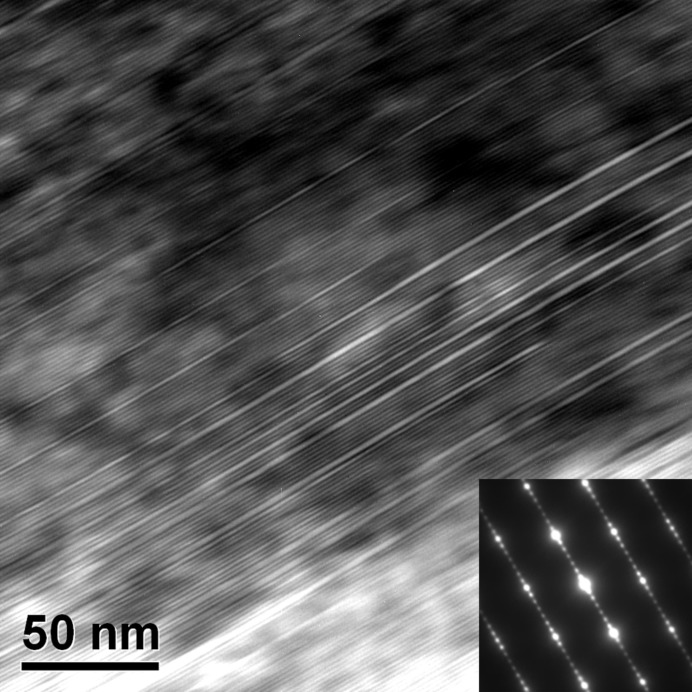
A TEM bright-field image along 〈2 1 0〉_7M_, showing the internal substructure of the 7M martensite plate. The inset shows the corresponding SAED pattern.

Mesoscopic observations [Figs. 3 ▸(*a*) and 4 ▸(*a*)] suggest that, at the local region of the austenite–martensite interface, the austenite is connected by only one martensite variant, which is quite different from the sandwich-structured martensite composed of two variants (Jin & Weng, 2002 ▸). Thus, close observation was made of the interior of a single martensite variant. Fig. 8 ▸ shows the TEM bright-field image obtained with the incident beam along 〈2 1 0〉_7M_, displaying the internal structure of the 7M martensite. The corresponding selected-area electron diffraction (SAED) pattern shown in the inset of Fig. 8 ▸ presents the typical character of 7M martensite in that six satellite spots exist between two fundamental spots. It is clearly seen from the figure that stacking faults parallel to the basal plane appear regularly in the interior of the 7M martensite. This indicates that the sandwich structure composed of two martensite variants in some other alloys is represented by a regularly faulted single martensite variant in the present alloy.

## 4. Discussion   

Since the martensitic transformation proceeds through coordinated atom displacement, lattice strain is a controlling factor for the transformation process. The lattice strains are cumulative as a function of the size of the formed martensite and become particularly pronounced at the austenite–martensite interface and at the martensite–martensite interfaces. By forming specially related variants, the lattice strains can be largely cancelled or self-accommodated. In fact, the self-accommodation of the transformation could be investigated in two aspects. The first is that the lattice misfits at the interfaces, both the martensite variant interfaces and the phase interfaces, should be minimal. The other is that the transformed volume should be largely compatible with its initial untransformed volume. For the former, the kinematic compatibility condition at the interfaces between martensite variants and between two phases defined by the geometrically nonlinear theory of martensitic transformation should be satisfied (Hane & Shield, 1998 ▸; James & Hane, 2000 ▸). For the latter, a minimum shape deformation during the transformation should be met. Thus, the specific configurations of the martensite group (diamond and spearheaded configuration) at different stages of growth in the present work should be the consequences of the two requirements.

### 4.1. Lattice deformation   

**Figure 9:**
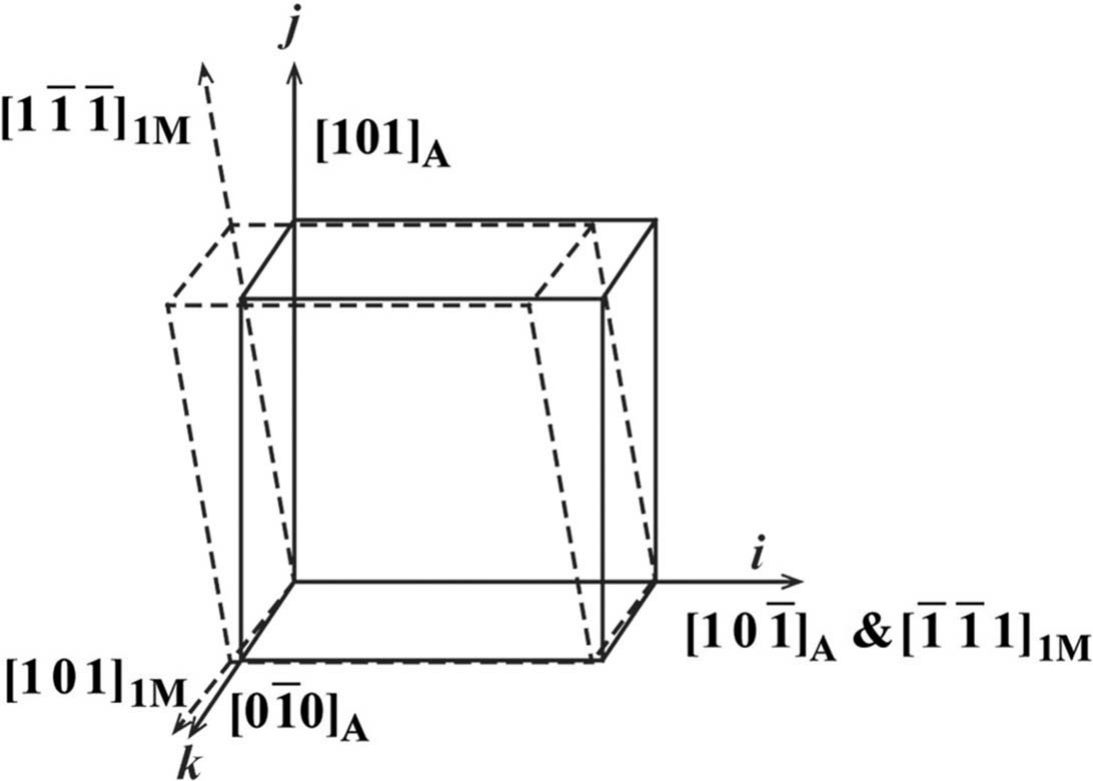
An illustration of the lattice deformation required to achieve the austenite to 7M martensite transformation under the Pitsch relation. The unit cell of austenite and the average unit cell of 7M martensite (referred to as 1M martensite) are outlined by solid lines and dashed lines, respectively.

To analyse the two aspects of self-accommodation, the lattice deformation for the martensitic transformation should be known. In the present work, the lattice deformation to change the austenite lattice to the 7M martensite lattice for the four variants in experimentally observed variant clusters is directly resolved from the orientation relationship (Li *et al.*, 2011*a* ▸), *i.e.* the Pitsch relation with {1 0 1}_A_||{1 −2 −10}_7M_ and 〈1 0 −1〉_A_||〈−10 −10 1〉_7M_, by examining the lattice correspondences between the two phases. For simplicity, the structural modulation of the 7M martensite is ignored. The superstructure of the 7M martensite is then reduced to an average monoclinic unit cell (referred to as 1M martensite) that corresponds to one cubic unit cell of the austenite lattice (Li *et al.*, 2011*a* ▸). The lattice deformation gradient can be deduced directly from the parallelisms of specific crystallographic planes and the directions of the two phases, as illustrated in Fig. 9 ▸.

Since the four variants in one variant group possess the same orientation for their {1 −2 −10}_7M_ planes and in-plane 〈−10 −10 1〉_7M_ directions, and these planes and directions are parallel to one of the {1 0 1}_A_ austenite planes and in-plane 〈1 0 −1〉_A_ directions, respectively, the orthonormal coordinate system along [1 0 −1]_A_–[1 0 1]_A_–[0 −1 0]_A_ can be used as the common coordinate system for the four variants in the variant group and the parent austenite, and thus to express the lattice deformation of the four variants. Under such a coordinate frame, the deformation gradient tensor ***M*** for the austenite to 7M martensite transformation can be expressed as
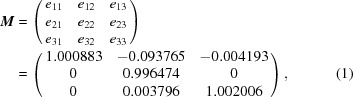
where the diagonal terms *e_ii_* represent an extension or contraction in the *i* direction and the off-diagonal terms *e_ij_* represent a shear along the *i* direction in the plane normal to the *j* direction. It is noted that the maximum shear lies in the *e*_12_ component, *i.e.* the (1 0 1)_A_[1 0 −1]_A_ system. Such a shear system should make the main contribution to the shape change of a single variant when transformed from the parent austenite.

The deformation gradient tensors of the four variants from the (1 0 1)_A_ group are constructed and displayed in Table 2 ▸. The deformation gradient tensors of the four variants are characterized by exactly the same values of the corresponding diagonal elements (principal strains) and the same absolute values of the corresponding off-diagonal elements (shear strains) but with permutation of the signs.

### 4.2. Kinematic compatibility of transformation twins and two phases   

Under the coordinate frame referring to the three cubic axes of the austenite, *i.e.* [1 0 0]_A_–[0 1 0]_A_–[0 0 1]_A_, the deformation gradient tensor ***M*** for the austenite to 7M transformation can be expressed as 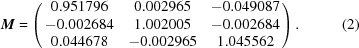
According to the polar decomposition theorem, the deformation gradient from austenite to 7M martensite under the coordinate frame of the austenite lattice can be written as ***M*** = ***RU***, where **R** is a rotation and **U** is a positive-definite symmetric matrix representing the transformation stretch. After such a manipulation, there are 12 distinct transformation stretches **U** for the present transformation from austenite to 7M martensite, which are equivalent to ‘cube-edge’ ones with a unique twofold axis along an edge of the original cubic unit cell (James & Hane, 2000 ▸). The compatibility condition for a twinned microstructure can be expressed as (Hane & Shield, 1998 ▸, 2000 ▸; James & Hane, 2000 ▸)
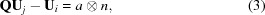
where **Q** is the twin rotation, *a* is the shear vector and *n* is parallel to the twinning plane normal. **U**_*i*_ and **U**_*j*_ represent the transformation stretch matrices.

A necessary and sufficient condition to solve the above equation is that one of the eigenvalues for the symmetry matrix **C** = 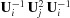 is equal to 1, *i.e.* λ_1_ ≤ λ_2_ = 1 ≤ λ_3_ (Hane & Shield, 1998 ▸, 2000 ▸; James & Hane, 2000 ▸). The solutions can be given by 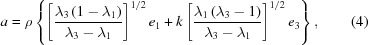


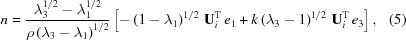
where *k* = ±1, ρ is chosen to normalize the twin plane normal *n*, and *e*_1_ and *e*_3_ are the eigenvectors corresponding to the eigenvalues λ_1_ and λ_3_, respectively. Through the proper combination of transformation stretch metrics, the condition for λ_2_ = 1 can be obtained and the corresponding solutions for the twinning equation are shown in Table 3 ▸. Thus, the misfit at the twin interfaces is minimal and the twin interfaces are achieved through energy minimization and strain compatibility.

The compatibility equation between austenite and a single variant can be expressed as (Hane & Shield, 1998 ▸, 2000 ▸; James & Hane, 2000 ▸)
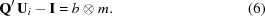
In this equation, the unknowns are the rotation **Q**′, the shape strain vector *b* and the habit plane normal *m*. Necessary and sufficient conditions to solve this equation are that the matrix 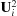 has ordered eigenvalues λ_1_ ≤ λ_2_ = 1 ≤ λ_3_. If these conditions are satisfied, the shape strain vector *b* and the habit plane normal *m* can be determined through the following equation (Hane & Shield, 1998 ▸, 2000 ▸; James & Hane, 2000 ▸)
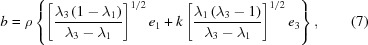


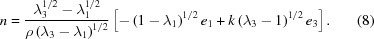
However, for the present 7M martensite, the eigenvalues of the matrix 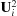 are determined to be λ_1_ = 0.9079, λ_2_ = 1.0036 and λ_3_ = 1.0960. It is impossible for an eigenvalue of the matrix 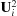 to be equal to 1.

In fact, the present 7M martensite plate contains certain numbers of stacking faults on the basal plane as the internal sub-structure, as shown in Fig. 8 ▸. It is generally accepted that such internal defects within the long-period stacking ordered structures are the reason that austenite and a single variant of martensite can coexist together (Zhu & Liew, 2003 ▸). Therefore, the stacking faults should be taken into account to achieve the compatibility of the phase interface.

Here, the internal faulted structure of 7M martensite is treated by introducing another variant and the austenite–two-variant model is considered. Since the internal stacking faults occur on the basal plane, the {0 0 20}_7M_ basal plane is chosen to be the possible twinning plane for the two martensite variants. Based on the minimum shear mechanism (Zhang *et al.*, 2010 ▸), the twinning elements for such a type of twin are resolved to be *K*_1_ = {0 0 20}_7M_, *K*_2_ = {2 0 0}_7M_, η_1_ = 〈1 0 0〉_7M_, η_2_ = 〈0 0 1〉_7M_ and *s* = 0.0873, corresponding to *K*_1_ = {0 −1 1}_A_, *K*_2_ = {0 1 1}_A_, η_1_ = 〈0 −1 −1〉_A_, η_2_ = 〈0 1 −1〉_A_ and *s* = 0.0873 within the framework of the parent austenite. Then, the compatibility conditions are given by the following equations (Hane & Shield, 1998 ▸, 2000 ▸; James & Hane, 2000 ▸; Ball & James, 1987 ▸):
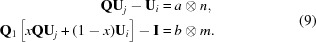
The volume fraction *x* can be resolved according to the following equation (Balandraud & Zanzotto, 2007 ▸):
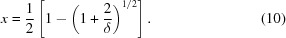
In this equation, δ can be determined from δ = 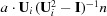. Calculation results show that the volume fraction *x* for the assumed variant is −0.0423. Normally, the volume fraction *x* should be between 0 and 1. The appearance of a negative volume fraction may indicate that the assumed twinning relation does not substantially exist, which thus confirms the nature of the internally defective structure for 7M martensite. Accordingly, the habit plane normal is calculated to be {0.720332, 0.692379, 0.041627}_A_, which is close to the experimentally determined one with a 2.1° deviation.

### 4.3. Self-accommodation of 7M martensite   

Generally, a single martensite variant cannot exist alone in the austenite–martensite microstructure. To meet the energy condition, the variants form a specific configuration to lower the transformation strain. Taking this as the starting point, we consider a group of four twin-related 7M variants (A, B, C and D) that originate from the (1 0 1)_A_ plane. The deformation gradient matrices for these variants were calculated under the orthonormal coordinate frame along [1 0 −1]_A_–[1 0 1]_A_–[0 −1 0]_A_, as given in Table 2 ▸.

Supposing an equal volume fraction for each variant in one variant group, then the total deformation gradient matrices for different twin variant pairs were calculated as follows.

For the type-I twin A:C pair,
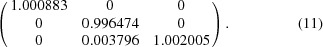
For the type-I twin B:D pair,
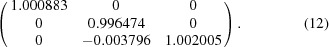
For the type-II twin A:B pair,
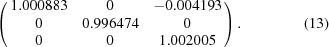
For the type-II twin C:D pair,
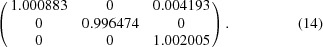
For the compound twin A:D pair,
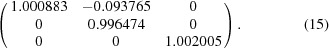
For the compound twin B:C pair,
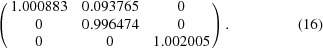
Since the four variants have the same values of the diagonal elements in the corresponding deformation gradient matrices, the principal strains cannot be cancelled by the combination of two variants in one variant group. On the other hand, in the total deformation gradient matrices of the type-I and type-II twin pairs, the maximum shear component (*e*_12_) can be totally cancelled. This indicates that both the type-I and type-II twin pairs are self-accommodated. However, for the compound twin pairs, the *e*_12_ component cannot be eliminated. In this context, the compound twin pairs cannot effectively accommodate the transformation strain.

Let us consider a single group of 7M martensite with four twin-related variants as a whole. If we assume that the four twin variants A, B, C and D have the same volume fraction, then the total deformation gradient matrix can be derived as
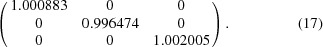
Here, all the shear components are eliminated by the combination of the four twin-related variants. Therefore, in the diamond microstructure of 7M martensite, the shear strain can be effectively accommodated when it grows from the parent austenite, whereas the principal strain from lattice elongation or contraction remains. Further accommodation of the transformation strain induced by lattice elongation or contraction can be achieved by the combination of different variant groups. As an example, the total deformation gradient matrix over all six variant groups from the same parent austenite was calculated under the assumption of equal volume for each variant group, *i.e.*

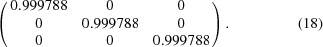



For this total deformation gradient matrix, all three principal components are almost equal to unity. This simple formulation is just provided to illustrate the possibility of self-accommodation among multi-groups of 7M martensite. For practical calculations, detailed information, including the shape, volume and configuration of twin variants in different groups, should be used as the input data.

### 4.4. Self-accommodation in martensite growth   

Based on the experimental observations, a possible route for the growth of diamond-like martensite into paired plates is proposed. At an early growth stage, the diamond-shaped martensite may expand through the extension of a type-I twin pair (A:C or B:D) or a compound twin pair (A:D or B:C). Since two variants with a compound twin relation are less self-accommodated, the extension of a compound twin pair would be less favoured. In contrast, the type-I twin combination can greatly minimize the shear strain. Thus, the extension of a type-I twin pair with a spearhead should be primarily responsible for the growth of the diamond-shaped martensite. Moreover, because the shear strains in a diamond group can be effectively compensated, the resistance against martensite growth mainly originates from lattice extension or contraction. According to the deformation gradient matrix of each variant shown in Table 2 ▸, the lattice distortion along [1 0 1]_A_ (perpendicular to the type-I twin interface in the diamond) is much higher than that of [0 −1 0]_A_ (perpendicular to the compound twin interface in the diamond). Thus, the lattice deformation along [1 0 1]_A_, *i.e.* extension through a compound twin, would encounter much higher resistance, which also indicates that the extension through a compound twin is less favourable.

When the diamond-shaped martensite further evolves into paired plates with spearheads, the extension of a type-I twin pair also plays a vital role. Let the plate-shaped variant combination be approximated by an ellipsoid with a shorter radius *y* and a longer radius *r*. According to the equation Δ*G*_el_ = *A*(*y*/*r*) where *A* is a constant (Ling & Owen, 1981 ▸), the larger the long radius *r*, the smaller the elastic strain energy change Δ*G*_el_. This indicates that the extension of a type-I twin pair can lower the elastic strain energy change Δ*G*_el_ during plate growth.

As the diamond-shaped martensite consists of only the type-I and compound twin systems, the type-II twin pair should be generated through further movement of the compound twin interface to accommodate the local transformation strain during the variant growth process. Locally, a compound twin interface has always appeared with its neighbouring type-I and type-II twin interfaces as a whole in the final self-accommodated plate-like microstructure.

## 5. Summary   

The microstructural characteristics associated with the austen­ite to 7M martensite transformation in an Ni_53_Mn_22_Ga_25_ alloy have been investigated using spatially resolved EBSD orientation characterization. The formation of diamond-shaped martensite with four variants (A, B, C and D) was shown at the initial nucleation stage of the martensitic transformation. Each diamond-shaped martensite consists of two type-I twin pairs (A:C and B:D) and two compound twin pairs (A:D and B:C), the long and short ridges of which correspond to a type-I twin interface and a compound twin interface, respectively.

The growth process of martensite contains two distinct steps. The first step refers to an isotropic expansion of the initial diamond-shaped martensite through the coordinated movement of the four side habit planes into the austenite matrix. The second step deals with an anisotropic elongation through the forward motion of a spearheaded type-I twin pair without thickening. At this stage, only one side habit plane of the type-I twin pair is activated, accompanied by the introduction of a type-II twin system through the motion of the twin interface between the two variants in compound twin and type-I twin relations.

TEM examinations demonstrate that the habit plane has a stepped structure, with {1 0 1}_A_ as the terrace plane and {0 1 0}_A_ as the step plane. The deformation gradient matrix of the martensitic transformation from austenite to 7M martensite was constructed according to the experimentally determined orientation relationship between the two phases.

Detailed crystallographic calculations show that locally, the compound twin pair (A:D or B:C) is less self-accommodated than the type-I twin pair (A:C or B:D) and the type-II twin pair (A:B or C:D). The shear strains can be effectively accommodated by grouping four twin-related variants, whereas the accommodation of principle strains induced by lattice elongation or contraction can be achieved by the combination of different variant groups.

## Figures and Tables

**Table 1:** Coordinates (*n*_1_, *n*_2_ and *n*_3_) of the calculated austenite–martensite interface plane normals and the mean interface plane values; the deviation (ω) of each interface plane normal from the mean value was also calculated and is listed in the table All the indices are expressed in the coordinate frame of the austenite lattice.

No.	*n*_1_	*n*_2_	*n*_3_	ω (°)
1	0.738894	0.665652	0.104611	2.10
2	0.738351	0.669307	0.082866	0.84
3	0.756754	0.650963	0.059750	1.82
4	0.723577	0.687154	0.065253	1.09
5	0.742925	0.662117	0.098304	1.85
6	0.733350	0.676126	0.071078	0.25
7	0.712261	0.698620	0.067933	1.99
8	0.762323	0.647162	0.006652	4.15
9	0.722641	0.688243	0.064122	1.18
10	0.724527	0.680720	0.108080	2.38
11	0.762845	0.644734	0.048838	2.52
12	0.728520	0.684741	0.019681	2.93
13	0.737620	0.666750	0.106587	2.20
14	0.745335	0.663200	0.068130	0.78
15	0.713322	0.697283	0.070484	1.90
16	0.727408	0.683697	0.058616	0.97
Mean value	0.736130	0.673329	0.068855	

**Table 2:** Deformation gradient tensors of four variants of the same group from the (1 0 1)_A_ plane [(1 0 1)_A_ group], represented in the frame referring to [1 0 −1]_A_–[1 0 1]_A_–[0 −1 0]_A_

Twin variant	Deformation matrix
A	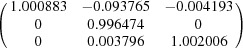
B	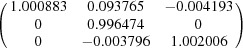
C	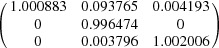
D	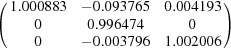

**Table 3:** Solutions of the twinning equation for the three types of twin in the present work

	Indices in cubic austenite			
Twinning type	*K*_1_	η_1_	*K*_2_	η_2_
Type I	{1 0 −1}_A_	Irrational	Irrational	〈−1 0 1〉_A_
Type II	Irrational	〈−1 0 1〉_A_	{1 0 −1}_A_	Irrational
Compound	{0 0 1}_A_	〈0 −1 0〉_A_	{0 1 0}_A_	〈0 0 −1〉_A_

## References

[bb1] Balandraud, X. & Zanzotto, G. (2007). *J. Mech. Phys. Solids*, **55**, 194–224.

[bb2] Ball, J. M. & James, R. D. (1987). *Arch. Ration. Mech. Anal.* **100**, 13–52.

[bb3] Bhattacharya, K. (1991). *Acta Metall. Mater.* **39**, 2431–2444.

[bb4] Bhattacharya, K. (2003). *Microstructure of Martensite: Why it Forms and How it Gives Rise to the Shape-Memory Effect.* Oxford University Press.

[bb5] Brown, P. J., Crangle, J., Kanomata, T., Matsumoto, M., Neumann, K. U., Ouladdiaf, B. & Ziebeck, K. R. A. (2002). *J. Phys. Condens. Matter*, **14**, 10159–10171.

[bb6] Buschbeck, J., Niemann, R., Heczko, O., Thomas, M., Schultz, L. & Fähler, S. (2009). *Acta Mater.* **57**, 2516–2526.

[bb7] Chai, Y. W., Kim, H. Y., Hosoda, H. & Miyazaki, S. (2009). *Acta Mater.* **57**, 4054–4064.

[bb8] Chernenko, V. A. (1999). *Scr. Mater.* **40**, 523–527.

[bb9] Chernenko, V. A., Seguí, C., Cesari, E., Pons, J. & Kokorin, V. V. (1998). *Phys. Rev. B*, **57**, 2659–2662.

[bb10] Chulist, R., Faryna, M. & Szczerba, M. J. (2016). *Acta Mater.* **103**, 836–843.

[bb11] Chulist, R., Pagounis, E., Böhm, A., Oertel, C. G. & Skrotzki, W. (2012). *Scr. Mater.* **67**, 364–367.

[bb12] Chulist, R., Straka, L., Lanska, N., Soroka, A., Sozinov, A. & Skrotzki, W. (2013). *Acta Mater.* **61**, 1913–1920.

[bb13] Chulist, R., Straka, L., Sozinov, A., Tokarski, T. & Skrotzki, W. (2017). *Acta Mater.* **128**, 113–119.

[bb14] Cong, D. Y., Zhang, Y. D., Wang, Y. D., Esling, C., Zhao, X. & Zuo, L. (2006). *J. Appl. Cryst.* **39**, 723–727.

[bb15] Cong, D. Y., Zhang, Y. D., Wang, Y. D., Humbert, M., Zhao, X., Watanabe, T., Zuo, L. & Esling, C. (2007). *Acta Mater.* **55**, 4731–4740.

[bb16] Gao, X., Huang, M. & Brinson, L. C. (2000). *Int. J. Plast.* **16**, 1345–1369.

[bb17] Hane, K. F. & Shield, T. W. (1998). *Philos. Mag. A*, **78**, 1215–1252.

[bb18] Hane, K. F. & Shield, T. W. (2000). *J. Elast.* **59**, 267–318.

[bb19] Hou, L., Dai, Y. C., Fautrelle, Y., Li, Z. B., Ren, Z. M., Esling, C. & Li, X. (2017). *Scr. Mater.* **134**, 85–90.

[bb20] Hou, L., Dai, Y. C., Fautrelle, Y., Li, Z. B., Ren, Z. M., Esling, C. & Li, X. (2018). *Scr. Mater.* **156**, 95–100.

[bb21] Inamura, T., Nishiura, T., Kawano, H., Hosoda, H. & Nishida, M. (2012). *Philos. Mag.* **92**, 2247–2263.

[bb22] James, R. D. & Hane, K. F. (2000). *Acta Mater.* **48**, 197–222.

[bb23] Jiang, C., Muhammad, Y., Deng, L., Wu, W. & Xu, H. (2004). *Acta Mater.* **52**, 2779–2785.

[bb24] Jin, Y. M. & Weng, G. J. (2002). *Acta Mater.* **50**, 2967–2987.

[bb25] Kainuma, R., Imano, Y., Ito, W., Sutou, Y., Morito, H., Okamoto, S., Kitakami, O., Oikawa, K., Fujita, A., Kanomata, T. & Ishida, K. (2006). *Nature*, **439**, 957–960. 10.1038/nature04493 16495995

[bb26] Karaca, H. E., Karaman, I., Basaran, B., Ren, Y., Chumlyakov, Y. I. & Maier, H. J. (2009). *Adv. Funct. Mater.* **19**, 983–998.

[bb27] Krishnan, M. (1998). *Acta Mater.* **46**, 1439–1457.

[bb28] Li, D., Li, Z. B., Yang, J. J., Li, Z. Z., Yang, B., Yan, H. L., Wang, D. H., Hou, L., Li, X., Zhang, Y. D., Esling, C., Zhao, X. & Zuo, L. (2019). *Scr. Mater.* **163**, 116–120.

[bb29] Li, Z. B., Dong, S. Y., Li, Z. Z., Yang, B., Liu, F., Sánchez-Valdés, C. F., Sánchez Llamazares, J. L., Zhang, Y. D., Esling, C., Zhao, X. & Zuo, L. (2019). *Scr. Mater.* **159**, 113–118.

[bb30] Li, Z., Jiang, Y., Li, Z., Sánchez Valdés, C. F., Sánchez Llamazares, J. L., Yang, B., Zhang, Y., Esling, C., Zhao, X. & Zuo, L. (2018). *IUCrJ*, **5**, 54–66. 10.1107/S2052252517016220 PMC5755577 29354271

[bb36] Li, Z. B., Xu, N., Zhang, Y., Esling, C., Raulot, J., Zhao, X. & Zuo, L. (2013). *Acta Mater.* **61**, 3858–3865.

[bb31] Li, Z. B., Yang, J. J., Li, D., Li, Z. Z., Yang, B., Yan, H. L., Sánchez–Valdés, C. F., Llamazares, J. L. S., Zhang, Y. D., Esling, C., Zhao, X. & Zuo, L. (2019). *Adv. Electron. Mater.* **5**, 1800845.

[bb32] Li, Z., Zhang, Y., Esling, C., Zhao, X., Wang, Y. & Zuo, L. (2010). *J. Appl. Cryst.* **43**, 617–622. 10.1107/S0021889810037180 PMC3253729 22477779

[bb33] Li, Z. B., Zhang, Y. D., Esling, C., Zhao, X. & Zuo, L. (2011*a*). *Acta Mater.* **59**, 2762–2772.

[bb34] Li, Z. B., Zhang, Y. D., Esling, C., Zhao, X. & Zuo, L. (2011*b*). *Acta Mater.* **59**, 3390–3397.

[bb35] Li, Z. B., Zhang, Y. D., Esling, C., Zhao, X. & Zuo, L. (2012). *Acta Mater.* **60**, 6982–6990.

[bb37] Li, Z. B., Zhang, Y. D., Sánchez-Valdés, C. F., Sánchez Llamazares, J. L., Esling, C., Zhao, X. & Zuo, L. (2014). *Appl. Phys. Lett.* **104**, 044101.

[bb38] Li, Z. B., Zou, N. F., Sánchez-Valdés, C. F., Sánchez Llamazares, J. L., Yang, B., Hu, Y., Zhang, Y. D., Esling, C., Zhao, X. & Zuo, L. (2016). *J. Phys. D Appl. Phys.* **49**, 025002.

[bb39] Ling, H. C. & Owen, W. S. (1981). *Acta Metall.* **29**, 1721–1736.

[bb40] Miyazaki, S., Otsuka, K. & Wayman, C. M. (1989). *Acta Metall.* **37**, 1873–1884.

[bb41] Murakami, Y., Otsuka, K., Hanada, S. & Watanabe, S. (1994). *Mater. Sci. Eng. A*, **189**, 191–199.

[bb42] Niemann, R., Backen, A., Kauffmann-Weiss, S., Behler, C., Rößler, U. K., Seiner, H., Heczko, O., Nielsch, K., Schultz, L. & Fähler, S. (2017). *Acta Mater.* **132**, 327–334.

[bb43] Nishida, M., Nishiura, T., Kawano, H. & Inamura, T. (2012*a*). *Philos. Mag.* **92**, 2215–2233.

[bb44] Nishida, M., Okunishi, E., Nishiura, T., Kawano, H., Inamura, T., Ii, S. & Hara, T. (2012*b*). *Philos. Mag.* **92**, 2234–2246.

[bb45] O’Handley, R. C., Murray, S. J., Marioni, M., Nembach, H. & Allen, S. M. (2000). *J. Appl. Phys.* **87**, 4712–4717.

[bb46] Otsuka, K. & Shimizu, K. (1974). *Trans. JIM*, **15**, 103–108.

[bb47] Pons, J., Chernenko, V. A., Santamarta, R. & Cesari, E. (2000). *Acta Mater.* **48**, 3027–3038.

[bb48] Righi, L., Albertini, F., Villa, E., Paoluzi, A., Calestani, G., Chernenko, V., Besseghini, S., Ritter, C. & Passaretti, F. (2008). *Acta Mater.* **56**, 4529–4535.

[bb49] Saburi, T. & Wayman, C. M. (1979). *Acta Metall.* **27**, 979–995.

[bb50] Scheerbaum, N., Lai, Y. W., Leisegang, T., Thomas, M., Liu, J., Khlopkov, K., McCord, J., Fähler, S., Träger, R., Meyer, D. C., Schultz, L. & Gutfleisch, O. (2010). *Acta Mater.* **58**, 4629–4638.

[bb51] Schroeder, T. A. & Wayman, C. M. (1977). *Acta Metall.* **25**, 1375–1391.

[bb52] Skokov, K. P., Khovaylo, V. V., Müller, K. H., Moore, J. D., Liu, J. & Gutfleisch, O. (2012). *J. Appl. Phys.* **111**, 07A910.

[bb53] Soejima, Y., Motomura, S., Mitsuhara, M., Inamura, T. & Nishida, M. (2016). *Acta Mater.* **103**, 352–360.

[bb54] Sozinov, A., Lanska, N., Soroka, A. & Zou, W. (2013). *Appl. Phys. Lett.* **102**, 021902.

[bb55] Stadler, S., Khan, M., Mitchell, J., Ali, N., Gomes, A. M., Dubenko, I., Takeuchi, A. Y. & Guimarães, A. P. (2006). *Appl. Phys. Lett.* **88**, 192511.

[bb56] Wayman, C. M. (1994). *Metall. Mater. Trans. A*, **25**, 1787–1795.

[bb57] Wechsler, D. S., Lieberman, D. S. & Read, T. A. (1953). *Trans. AIME* **197**, 1503–1515.

[bb58] Wu, S. K. & Yang, S. T. (2003). *Mater. Lett.* **57**, 4291–4296.

[bb59] Ye, F. & Zhang, W. Z. (2002). *Acta Mater.* **50**, 2761–2777.

[bb60] Zhang, C., Zhang, Y., Esling, C., Zhao, X. & Zuo, L. (2017). *IUCrJ*, **4**, 700–709. 10.1107/S2052252517011332 PMC5619861 28989725

[bb61] Zhang, Y. D., Esling, C., Zhao, X. & Zuo, L. (2007). *J. Appl. Cryst.* **40**, 436–440.

[bb62] Zhang, Y., Li, Z., Esling, C., Muller, J., Zhao, X. & Zuo, L. (2010). *J. Appl. Cryst.* **43**, 1426–1430. 10.1107/S0021889810037180 PMC3253729 22477779

[bb63] Zhao, X. Y., Wen, J. H., Gong, Y. Y., Ma, S. C., Hu, Q. B. & Wang, D. H. (2019). *Scr. Mater.* **167**, 41–45.

[bb64] Zhu, J. J. & Liew, K. M. (2003). *Acta Mater.* **51**, 2443–2456.

